# Whole Genome Gene Expression Analysis Reveals Casiopeína-Induced Apoptosis Pathways

**DOI:** 10.1371/journal.pone.0054664

**Published:** 2013-01-31

**Authors:** Alejandra Idan Valencia-Cruz, Laura I. Uribe-Figueroa, Rodrigo Galindo-Murillo, Karol Baca-López, Anllely G. Gutiérrez, Adriana Vázquez-Aguirre, Lena Ruiz-Azuara, Enrique Hernández-Lemus, Carmen Mejía

**Affiliations:** 1 Computational Genomics Department, National Institute of Genomic Medicine, México City, México; 2 Microarray Core Facility, National Institute of Genomic Medicine, México City, México; 3 Chemical Physics Department, Institute of Chemistry, National Autonomous University of México, México City, México; 4 Medicinal Chemistry Department, College of Pharmacy, University of Utah, Salt Lake City, Utah, United States of America; 5 School of Sciences, Autonomous University of the State of México, Toluca, México; 6 Genomic Medicine and Environmental Toxicology Department, Institute for Biomedical Research, National Autonomous University of México, México City, México; 7 Nuclear and Inorganic Chemistry Department, Chemistry School, National Autonomous University of México, México City, México; 8 Center for Complexity Sciences, National Autonomous University of México, México City, México; University of California San Francisco, United States of America

## Abstract

Copper-based chemotherapeutic compounds Casiopeínas, have been presented as able to promote selective programmed cell death in cancer cells, thus being proper candidates for targeted cancer therapy. DNA fragmentation and apoptosis–in a process mediated by reactive oxygen species–for a number of tumor cells, have been argued to be the main mechanisms. However, a detailed functional mechanism (a model) is still to be defined and interrogated for a wide variety of cellular conditions before establishing settings and parameters needed for their wide clinical application. In order to shorten the gap in this respect, we present a model proposal centered in the role played by intrinsic (or mitochondrial) apoptosis triggered by oxidative stress caused by the chemotherapeutic agent. This model has been inferred based on genome wide expression profiling in cervix cancer (HeLa) cells, as well as statistical and computational tests, validated *via* functional experiments (both in the same HeLa cells and also in a Neuroblastoma model, the CHP-212 cell line) and assessed by means of data mining studies.

## Introduction

Basic research in cancer has lead us to attain remarkable advances in our understanding of both, cancer cell biology and cancer genetics. One of the key concepts developed was the fact that apoptosis and the genes involved in its triggering and control may have a determinant effect on the malignant phenotype. Defective apoptosis mechanisms are major causative factors in tumor development and progression. Oncogenic mutations disrupting apoptosis are known to play a role in tumor initiation [1], but also in progression and metastasis. Also, we have strong evidence pointing-out to other oncogenic changes that promote apoptosis, hence leading the cells to override apoptosis during multistage carcinogenesis. As is the case, the most cytotoxic anticancer agents are the ones able to induce apoptosis. This brings on, the riveting possibility that defects in apoptotic programs contribute to treatment failure. Since the same mutations that suppress apoptosis during tumor development also contribute to the reduction of treatment sensitivity, apoptosis may provide a conceptual framework associating cancer genetics and cancer therapy [2]. Our current understanding of the complexities of apoptosis and the mechanisms developed by tumor cells to resist engagement of cell death has focused research effort into the design of strategies to induce apoptosis selectively in cancer cells [1]–[3].

Cervix-uterine carcinoma (CUC) is the second most common type of cancer among the female population in the third world. The main risk factors associated with CUC include age (25–64 years), low cultural, social and economic levels; multiple sexual partners and Human Papilloma Virus (HPV) infection by types 16, 18, 31, 33, 35, 39, 45, 52, 56, 58 and 59. CUC has several stages including carcinoma *in situ* (stage 0), microscopic cancer without dissemination (stage I), low and medium proliferative stances (stages II and III) and stage IV characterized by metastasis to bladder or rectum (A) or distal parts as lungs (B) [4]. The elective treatment includes different schemes that go from prophylactic prevention by vaccines, to hysterectomy, exenteration, electrosurgical excision or LEEP, undergoing through radiotherapy or chemotherapy. Commonly, chemotherapy comprises 5 fluorouracil (5-FU), cisplatin, carboplatin, iphosphamide, paclitaxel and cyclophosphamide. Due to the common occurrence of CUC, HeLa, a cell line derived from cervix cancer, is considered an adequate model for neoplasic transformations and also for studies on chemotherapy [5].

**Figure 1:**
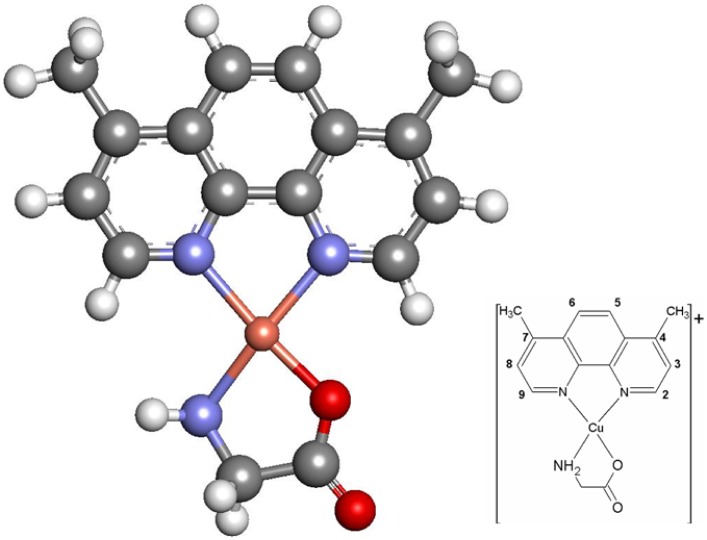
Structure of the Casiopeína II-gly molecule. Main figure depicts a full-atom quasi-3D rendering with optimized geometry. Grey atoms are Carbon, blue ones are Nitrogen, red ones are Oxygen, white are Hydrogen and the orange one is Copper as it can be seen at the inset.

Casiopeína II-gly (Cas II-gly), or [Cu(4,7-dimethyl-1,10 phenanthroline)(glycinate)]NO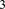 (for details of the chemical structure of Cass II-gly see Figure 1), is a recently developed Copper-containing-drug that has shown as a promising chemotherapeutic agent on tumor models as well as in experimental protocols [6]–[9]. Its action mechanism is not completely elucidated. However, it has been suggested that it can act by means of (1) direct interaction with DNA through intercalation or adduct formation [10], (2) mitochondrial toxicity [7], [11]–[13] and/or (3) oxidative damage after ROS generation [6], [14]–[18]. Since functional mechanisms of Cas in cervical cancer have not been analyzed yet, this study further explores the effects of Cas [14] inducing mitochondrial apoptosis and oxidative stress in HeLa cells. As we will further discuss, Cas induced apoptosis by over-production of ROS and concomitant decrease in intracellular levels of GSH. In order to compare our results in relation with the potential effect of Casiopeínas in tumor cells, we will recall the case of Neuroblastoma [18].

## Materials and Methods

### Synthesis of Antineoplasic Drug: Casiopeínas

Casiopeína II-gly (Fig. 1) was synthesized as previously described and dissolved in sterile water [19].

### Treatments and Cellular Viability

HeLa cervix-uterine cell line and neuroblastoma cell line CHP-212 (American Type Culture Collection, Rockville, MD, USA) were maintained at 37°C in 5% CO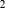 under sterile conditions in Dulbecco’s modified Eagle medium (DMEM, Sigma), supplemented with 10% fetal bovine serum (Sigma) and F-12 medium (for CHP). Cells were treated with Cas II-gly in 96-well microplates during 6 hours (HeLa) and 2 hours (CHP-212). Viability determination tests were performed with sulforhodamine-B dy: absorbance was quantified in a microtiter plate reader at 560 nm (Lab-system Uniskan) [20]. After establishing a first limit, viability was determined with a Neubauer’s camera by quadruplicated(two independent observers). Half-maximal inhibitory concentrations (IC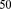)were used as working concentrations (40 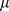
M in the case of HeLa and 14 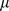
M for CHP-212).

### Whole Genome Gene Expression Analysis in HeLa Cells

HeLa cells whole genome gene expression experiments (double-triplicates for cases/controls) were performed in total mRNA extracted under the GPL570 which is based on the Affymetrix HGU133Plus 2 microarray GeneChip platform [21]. Affymetrix HG-U133-Plus2 has 54,675 probe sets, each consisting of eleven 25-mer probes. Each probe set is designated by an Affymetrix ID together with a GenBank accession ID. It includes 604,258 probe sequences.

### Determination of Apoptosis

Apoptotic activity was measured by expression quantification of different proteins in cytoplasmic fractions by Western blot, previous subcellular fractionation with Mitochondria/Cytosol Fractionation Kit (CALBIOCHEM). Briefly, 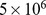 control and treated cells were harvested, washed twice with ice-cold PBS, resuspended in cytosol extraction buffer mix plus 1 M DTT and protease inhibitors cocktail (all of them from CALBIOCHEM). Protein concentrations of each sample were determined using Bio-Rad’s DC Protein Assay kit II and a microtiter plate reader at 630 nm. Samples containing 10 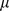
g/ml of protein were mixed with an equal volume of 2X sample buffer (125 mM Tris-HCl, pH 6.8, 20% glycerol, 4% SDS, 0.02% bromophenol blue, and 10% 2-mercaptoethanol) and boiled during 5 min. Samples were cooled on ice for 5 min, centrifuged for a short time, and exposed to 10% SDS polyacrylamide gel electrophoresis (PAGE). Proteins were transferred to a nitrocellulose membrane for 45 min at 500 mA with 25 mM Tris-HCl, pH 8.0, 195 mM glycine, and 20% methanol. The membrane was blocked with 2% light milk in 1X PBS overnight, washed three times in 1X PBS and 0.2% Tween 20, and caspase-3 primary antibody (Santa Cruz Biotechnology) was added for 24 h at 4°C. After three consecutive washes in 1X PBS and 0.2% Tween 20, the membrane was incubated with a donkey antigoat IgG-horseradish peroxidase complex (Santa Cruz Biotechnology) during 1 h at room temperature, followed by three washes with 1X PBS and 0.2% Tween 20. Then, chemiluminescence was visualized using the SuperSignal West Dura Extended Duration Substrate kit (Thermo Scientific). The blot was exposed to Kodak XAR-5-ray film (Sigma) for 5 min and then revealed. Based on previously expressed *caspase-3* protein measurements in a time-course for CHP-212 cells [18], the following determinations were made at 2 h; HeLa cells were treated for 6 h according previous reports [19]. A similar procedure was used for *caspase-8*, *cyt C*, *Bcl-2*, *Bax* and 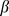-*tubulin* (Santa Cruz Biotechnology). Band intensities were quantified using ImageJ software version 1.41e. Disruption of the mitochondrial transmembrane potential (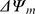)was also measured to determine the presence of apoptosis. Both cell lines were incubated with 50 nm of MitoTracker 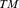 GreenCmxROS (Molecular 7 Probes) during 20 min at 37°C. After that, fluorescence was analyzed in a FACScan flow cytometer (Becton Dickinson Biosciences).

### Evaluation of Reactive Oxygen Species and Glutathione

In all cells, hydrogen peroxide (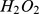) and superoxide (O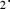) levels were determined. Quantification of H
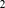
O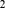 was made using AmplexRed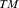 which is a non-colored substrate that reacts stoichiometrically (1∶1) with hydrogen peroxide generating resorfurin, a substance that can be detected by flow cytometry. 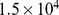 cells/ml, were washed up with KRPG as buffer and were then incubated with the reaction mixture (50
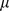
M AmplexRed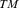 plus 0.1U/ml HRP) for 30 min in darkness. Meanwhile, detection of superoxide (O2-) was performed by means of MitoSox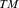, an analog of hydroethidine. Cells were incubated with 250 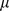
l of MitoSox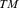 diluted in DMSO (stock solution 5 mM), by 10 min in darkness at 37°C. Both determinations were performed using cells UV-irradiated for three hours as a positive control. The oxidation products were detected using the FL2-H channel of a FACScan flow cytometer (Becton Dickinson Biosciences). Since low levels of glutathione are observed in early stages of apoptosis, we measured glutathione by Glutathione kit (Calbiochem, CA) with monochlorobimane (MCB) that forms a GSH adduct by action of the glutathione-S-transferase (GST). Cellular pellets of HeLa and CHP-212 were resuspended in Cell Lysis Buffer supplemented with 25 ml of 4
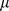
M of MCB plus 50 U/ml GST. Negative control was MCB plus Cell Lysis Buffer. All samples were incubated for15–30 min at 37°C and total number of cells were counted in a microtiter plate reader at 380/460 nm.

### Quantitative PCR Validation of Selected Genes

#### RNA extraction and preparation

In order to extract RNA, HeLa cells (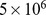 cells) treated (or not) with 40 mM Cas II-gly for 6 hours were collected in 1 ml of Trizol (Invitrogen) and extraction was performed according to the manufacturer instructions. RNA integrity was determined by means of gel electrophoresis, and nucleic acid concentration was measured with a spectrophotometer (Amersham Pharmacia Biotech). Reverse transcription was performed with Superscript II Reverse Transcriptase (Invitrogen, No. cat. 18064-014) and cDNA was amplified with Taq DNA Polymerase (Invitrogen, No. cat. 11615-010). PolyA+ RNA from HeLa cells were selected by affinity chromatography using an oligo (dT) cellulose column.

#### Real-Time quantitative RT-PCR (qRT-PCR)

Expression levels of apoptotic molecules (caspase-3, caspase-8, cyt C, bcl-2 and bax), were determined for the RNA isolated from treated and control HeLa cells. cDNA was synthesized and pooled into microtiter wells containing cDNA obtained from 100 mg of total RNA. To determine the relationship between cycle number (Ct) and expression of each mRNA subunit, specific calibration was made by using serial dilutions of cDNA. In all cases, data from cDNA samples was collected and each amplification was carried out in duplicate. Reactions were performed with TAQurate GREEN Real Time PCR Master Mix enzyme (EPICENTRE TECHNOLOGIES, No. cat. TM046400), using HPRT1 as a standard control. PCR amplifications were generated in a Rotor-Gene Q-2plex HRM System (QIAGEN). Negative controls (no reverse transcription) consistently showed no increase in fluorescence. As a reference control, we used the sum of values obtained from all subunits under examination; data are presented as number of copies of mRNA of each apoptotic molecule.

### Integrated Analysis

A foundational paradigm in contemporary genomic research is data integration (DI). The main objective of DI is that of *making sense* out of the extremely large datasets in, for instance, genome-wide expression analyses. With the upcoming of new techniques in high throughput molecular biology, maybe just one thing has been established: complex biological systems need to be studied from several points of view, in order to unveil the actual molecular mechanisms responsible for their functions. In the present case, our aim is to sketch some hints for a proposal of functional mechanisms behind gene expression in cancer and, in particular regarding the action of pharmacological targets for chemotherapeutic agents like, for instance Copper-based compounds (Casiopeínas). In order to pursue such a goal, we performed whole genome gene expression analysis, gene-specific expression measurement, flow cytometry experiments, as well as selected protein determination by western blots, and also statistical and computational analysis of the associated biological pathways/processes as we will describe in what follows.

### Differential Expression Analysis

After pre-processing (background correction, followed by normalization and *summarization*) of the samples [22] according with the well known RMA algorithm [23], a statistical analysis was implemented by using an algorithm based on linear models (limma). The methodology is intended to look up for significant differentially expressed genes (full expression data matrix may be available upon request). Empirical Bayes and other shrinkage methods are used to borrow information across genes making the analyses stable even for experiments with a relatively small number of arrays. This method hence allows for very general experiment classes to be analyzed as easily as one may do with single replica experiments. The approach relies on two matrices, the first one is called the *design matrix* which gives a representation of the different RNA targets which have been hybridized to the arrays. The second one, or *contrast matrix*, allows the coefficients defined by the design matrix to be combined into contrasts of interest. Each contrast corresponds to a comparison of interest [24].

### Statistical Enrichment Analysis for GO Categories, Biochemical Pathways and Selected Gene Sets

We performed a Gene Ontology (GO) [25] enrichment analysis consisting in the determination of statistical over-representation of GO categories within our set of significant genes. Significance assessment was made by means of ‘urn model’ hypergeometric distribution tests. Testing a GO term amounts to drawing the genes annotated at it from the ‘urn’ (gene universe) and classifying them as to whether they belong to a certain GO category. By counting and calculating the resulting proportions it is possible to perform a significance test, namely, an hypergeometric test (which is known to be equivalent to a one-tailed Fisher’s Exact test). In order to correct for multiple testing Benjamini-Hochberg [26] algorithm correction -the so called False Discovery Rate (FDR)- was used. Only associations whose corrected p-values ranged below 0.05 were considered significant. GO enrichment analysis was performed by mapping common functional themes for a given gene set on the GO hierarchy, and considering the output of this mapping as a graph. Gene sets were selected from a list of genes significantly up-regulated in a microarray experiments. The main advantage of this method is that it can be used directly and interactively on molecular interaction graphs, allowing to take full advantage of the hierarchical tree organization of the GO database.

In the other hand, Reactome [27] biological/biochemical pathway over-representation analysis was performed to determine the molecular pathways in which gene IDs in our list were strongly enriched. Reactome is a an open-source, open access, manually curated and peer-reviewed pathway database that may help to understand the biological context of genomic data. Significance assessment was also made by means of ‘urn model’ hypergeometric distribution tests.

We also tested our whole genome gene expression data with Ingenuity ® an integrative biomolecular tool for analysis for complex ’omics data [28]. We used the Ingenuity Pathway Analysis (IPA) module. IPA searches the Ingenuity Knowledge Base (IKB), the largest database housing biological and chemical relationships extracted from the scientific literature, by means of a proprietary bioinformatics tool make possible to interpret an experimental gene expression dataset in the context of biological processes, pathways, and molecular networks. We focused, in particular, in the biological processes affected, as well as in the implicit biochemical signaling pathways. Fisher’s exact test is performed to calculate the associated p-value, taking into account both, the number of molecules in the dataset that participate in some biological function or pathway, and the total number of molecules known to be associated with that function or pathway in the IKB. p-values less than 0.05 indicate a statistically significant, non-random association between a set of molecules in the dataset and a given biological feature.

Previous analysis in this work were mostly derived from the list of differentially expressed genes i.e. single-gene analysis. In order to include the collective behavior of genes within pathways and functional modules, we performed Gene Set Enrichment Analysis (GSEA) to determine whether members of gene sets 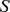 tend to occur at the top or bottom within a ranked list 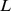 (genes showing largest difference between phenotypes) [29], [30]. GSEA was then applied to our expression data set considering the Cancer Modules (CM) sub-collection. Relevant parameters used for these sub-collections were the following: *permutations* - 1000, *scoring scheme* - weighted and *metric* - Signal2Noise.

### Statistical and Computational Tools

Microarray pre-processing of the data was performed by using the affy library in BioConductor running under [R] on a 128 Gb RAM 8-Power5+ dual core-processor, symmetric multiprocessing (SMP) unit by IBM. Whereas all statistical tests were performed on a Dell Precision Series 8 Gb RAM QuadCore Workstation by using limma package in [R]/BioConductor. Graphical depiction was performed with Cytoscape. Other calculations and analyses were performed with custom [R] and shell scripts. Pathway enrichment analysis was made by means of hypergeometric testing of databases by Reactome [27]. Gene Set Enrichment Analyses were performed with the GSEA Java library [30].

Functional validation experimental data for the Western blots, flow cytometry for ROS and Glutathione measurement by ELISA, are presented as means 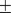 standard error. For GSH levels measurements, results were reported from experimental groups consisting of three wells and experiments replicated once. All determinations were developed by triplicate and differences between groups were analyzed by ANOVA analysis where significance was preset at 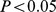. Statistical analysis for flow cytometry in apoptosis and ROS determination was performed with FlowJo.

## Results

### Whole Genome Gene Expression Analysis

**Figure 2:**
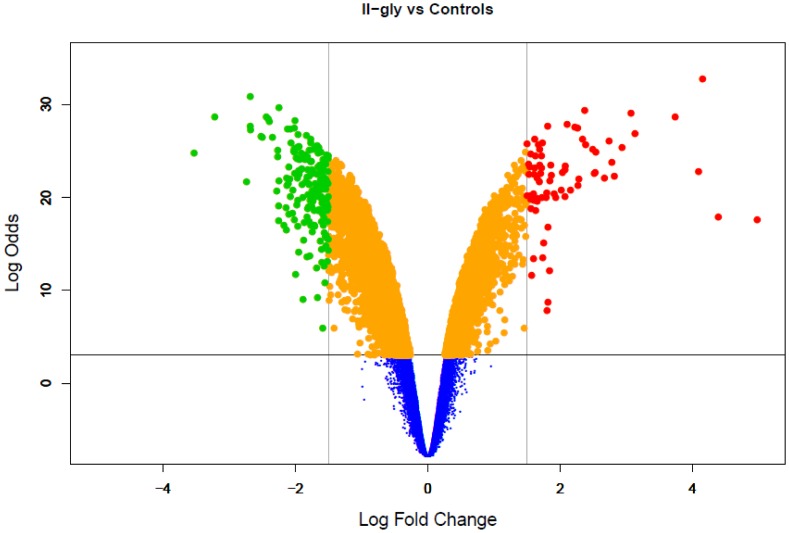
Volcano Plot for differential gene expression. HeLa Cells treated with Casiopeína II-gly vs untreated HeLa cells as Controls. Scattered points represent genes: the x-axis is the 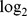 fold change for the ratio Cas-II-gly treated cells vs untreated cells, whereas the y-axis is the 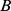-statistic or *Log Odds*, 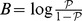 where 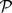 is the probability that a gene has statistical significance in its differential expression. Red dots are thus genes significantly over-expressed after treatment with CAS, and green dots are genes significantly under-regulated after treatment.

**Figure 3:**
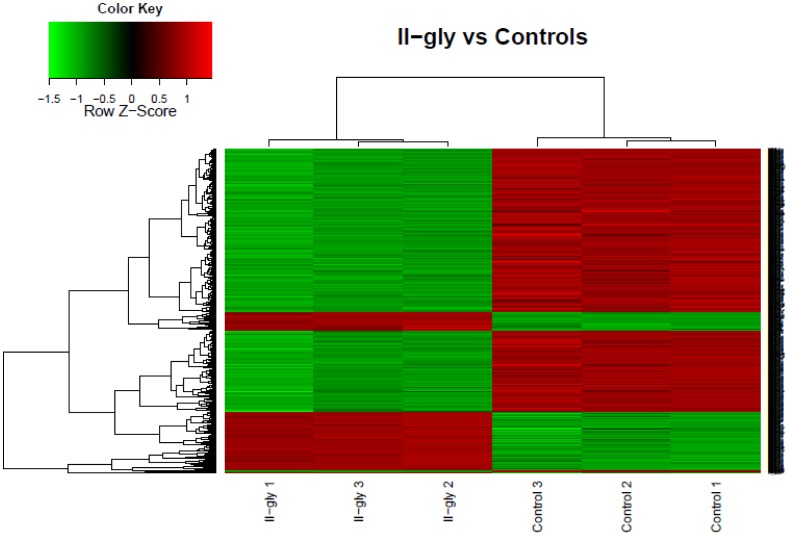
HeatMap for hierarchical clustering of differential gene expression. HeLa Cells treated with Casiopeína II-gly vs untreated HeLa cells as Controls. We can notice the clear presence of groups of genes that are systematically, either up-regulated (red) or down-regulated (green) in treatment *versus* controls.

For the whole genome gene expression experiments in HeLa we used the Affymetrix HGU 133 plus 2.0 Array. Differential expression analysis for HeLa cells treated with Casiopeína II-gly *versus* untreated HeLa cells as controls lead us to 1037 differentially expressed genes and isoforms that resulted statistically significant under our imposed parameters (Experimental data has been deposited in GEO [31] and is publicly available under the accesion key: GSE41827 [32]). The threshold was set by requiring a value for the *Log Odds* or 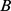-statistic, 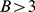 which corresponds approximately to a p-value lower than 0.05 (Figure 2). The 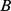-statistic is defined as 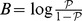 where 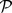 is the probability that a gene has statistical significance in its differential expression. A value of 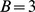 then implies 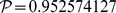 (representing a p-value of 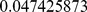). For the referred gene expression experiments we have a 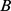-statistic empirical distribution ranging as follows 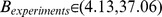. As expected, groups of samples/genes clustered together in an unsupervised hierarchic analysis corresponding to phenotypes and genes with similar expression profiles [33], respectively (Figure 3). Figure 3 corresponds to the contrast between treated and untreated HeLa Cells with Casiopeina II-gly. Hierarchical clustering was performed, both among samples and gene expression levels. Similar patterns among samples and/or experimental conditions are clustered and a dendrogram on top is drawn. Additionally, a dendrogram added to the left side clusters genes that show similar expression patterns across number of samples (average gene expression values), thus we can see that genes with similar expression patterns are consistent within case/control samples. The full database of differentially expressed genes (DEG-DB) with their corresponding statistics and expression levels is available at the following webpage: http://genomicacomputacional.inmegen.gob.mx/ehernandez/Casio/Casio_report.html.

When looking at the DEG-DB, results quite interesting to notice that the genes with better statistics (DEG-DB is sorted in decreasing order of the respective 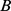-statistic) correspond to the upregulation of molecules activated in response to cellular stress (specially oxidative stress). Such is the case of the 70 kD heat shock protein *HSP6* (log fold change (LFC) = 6.64), of the heme oxygenase *HMOX1* (LFC = 4.18)and likely, of the Tribbles homolog 3 (induced by NF-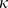
B)(LFC = 2.38). Another well-ranked overexpressed molecule is the zinc transporter *SLC30A1* that is known to participate in detoxification processes [34]. In the other hand, molecules involved in cell cycle such as *FBXO5* and *MYC* (that also plays an important role in apoptosis and cellular transformation) appear down regulated [35].

In line with the hypothesis of intrinsic apoptosis activated by oxidative stress, we can notice over-expression of *BCL-10*(LFC = 2.15), that activates *caspase-9* and interacts with *TNF*; as well as a over-expression of *NDRG1* which is a *p53*-mediated caspase activator [36]. In contrast, molecules associated with the extrinsic route to apoptosis appear under-expressed, this is the case of the death-domain receptor *FADD*(LFC = −2.67), that together with over expression of *MOAP1* (that interacts with the death-domain receptor and drive caspase-8 and -10 to the nucleus) and *DEDD2* may imply an overall reduction of the activity of *TNFR1* signaling [37], hence low activity of the apoptotic extrinsic pathway. The gene expression pattern also points out to cell cycle stop features: the combined effect of *ATF3* upregulation (LFC = 1.79), *CDKN1A* over expression together with *CCNE-1* and *TUSC2* down regulation, may cause the cell cycle to stack between G1 and S1 phases; whereas down-regulation of *FBXO5*(LFC = −2.68) and *ERCC6L*(LFC = −1.29) may arrest the cell cycle in the M-phase [38]. With regards to oxidative stress as a mechanism for triggering intrinsic apoptosis, we can notice over-expression of *HMOX1*, combined with over-expression of *GADD45G* (which regulates stress by activating the p38/JNKMTK1/MEKK4/kinase pathway) [39], up-regulation of *MT1X* (that regulates metals and molecules with free radicals); and also with low-expression of metal transporters (in particular Copper) *SLC11A2* and *SCO2*.

The DEG-DB also shows genes implicated in DNA damage/regeneration with riveting patterns: in the one hand, *ID2/ID2B* presents high expression level and *H2AFX* is down regulated, trademarks of DNA damage response. In the other hand we have over-expression of *HIST1/H2BG* (implied in DNA stability, transcription, repair and replication) and *GADD45B* (which activates the apoptotic cascade in response to DNA damage), as well as over-expression of *RBM4* (DNA repair) and *GADD45A*(cell cycle regulation after DNA damage). *p53* activation process leading to apoptosis seem to be present since there is also over-expression of both *PPP1R15A*, and *PPP1R3C* that are responsible of the phosphorylation of *TP53* in the Ser-15 site driving the activation of *p53* and thus forming of *p21* leading to cell cycle arrest and apoptosis.

### Statistical Enrichment Analysis

In order to reveal biological function alterations behind the differential gene expression patterns, data mining and statistical assessment techniques have been implemented. The rationale behind such methods is that if a number of molecules, known to be involved in a certain process or pathway, appear deregulated; then it is likely that the processes behind are functioning also abnormally. Statistical significance is usually assessed by comparing the number of *hits* on a given category or pathway *versus* that expected from a random sorting of these genes.

#### Gene ontology

Gene Ontology enrichment look up was performed by mapping the predominant functional themes of the differentially expressed gene set, for HeLa cells treated with Cas II-gly *versus* untreated cells, on the GO Biological Processes (BP) hierarchy. Some selected categories (relevant to this study) along with their respective statistics, can be seen in Table 1. For the complete data table (comprising 165 categories) please refer to the website: http://genomicacomputacional.inmegen.gob.mx/ehernandez/Casio/Casio_GO.xls. We can notice that biological processes related with programmed cell death (specially apoptosis), cell cycle arrest, MAP3K activity, response to stress, mitochondrial membrane permeability, caspase activation (in particular, the one triggered by *cyt C*) and DNA damage response are all significatively enriched within our differential expression gene set. As it was already mentioned regarding differential expression, this pattern is consistent with the hypothesis of Cas II-gly being involved in the development of cellular stress by ROS leading to DNA damage response as well as the activation of MAPKKK signaling. Caspase activation and mitochondrial membrane anomalies may also lead to cell cycle arrest and apoptosis.

**Table 1:** Statistically Significant Enrichment of Gene Ontology Categories for differentially expressed genes in HeLa cells treated with Cas II-Gly.

GO-ID	FDR-corrected p-value	Genes in test set
16265	3.85E-07	death
8219	3.85E-07	cell death
6915	1.00E-06	apoptosis
12501	1.00E-06	programmed cell death
42981	4.88E-05	regulation of apoptosis
43067	4.88E-05	regulation of programmed cell death
7050	6.19E-05	cell cycle arrest
8632	7.96E-05	apoptotic program
22402	1.08E-04	cell cycle process
6917	1.08E-04	induction of apoptosis
12502	1.08E-04	induction of programmed cell death
6950	1.20E-04	response to stress
6974	1.20E-04	response to DNA damage stimulus
43065	2.94E-04	positive regulation of apoptosis
43068	2.98E-04	positive regulation of programmed cell death
7049	6.50E-04	cell cycle
185	8.90E-04	activation of MAPKKK activity
46902	8.90E-04	regulation of mitochondrial membrane permeability
8629	9.46E-04	induction of apoptosis by intracellular signals
8625	3.19E-03	induction of apoptosis via death domain receptors
8630	7.99E-03	DNA damage response, signal transduction resulting in induction of apoptosis
30262	8.29E-03	apoptotic nuclear changes
7006	8.29E-03	mitochondrial membrane organization and biogenesis
8633	8.29E-03	activation of pro-apoptotic gene products
43123	9.35E-03	positive regulation of I-kappaB kinase/NF-kappaB cascade
8624	1.84E-02	induction of apoptosis by extracellular signals
6919	1.86E-02	caspase activation
43280	2.03E-02	positive regulation of caspase activity
42770	2.03E-02	DNA damage response, signal transduction
43618	2.57E-02	regulation of transcription from RNA polymerase II promoter in response to stress
43619	2.57E-02	regulation of transcription from RNA polymerase II promoter in response to oxidative stress
43620	2.57E-02	regulation of transcription in response to stress
41	3.28E-02	transition metal ion transport
43066	3.45E-02	negative regulation of apoptosis
43069	3.56E-02	negative regulation of programmed cell death
51881	4.12E-02	regulation of mitochondrial membrane potential
8635	4.12E-02	caspase activation via cytochrome c
279	4.70E-02	M phase
42771	4.97E-02	DNA damage resp., signal transduction by p53 class mediator; induction of apoptosis

#### Reactome

In Table 2 we present the results for the statistical enrichment analysis for molecules in relevant biochemical pathways within the Reactome database [27]. All the statistically significant pathways (i.e. those with FDR corrected p-value 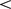 0.05) are presented. As in the case of GO-BP analysis: *TNF* and Death receptor signaling, response to metal toxicity, cell cycle arrest and apoptosis are over-represented. In a similar fashion, these are in line with the mechanistic hypothesis of Cas II-gly triggering cell death by apoptosis by means of response to oxidative stress (caused by metal-induced ROS) and cell cycle arrest. *TNF* signaling, for instance, belongs to a sub-network which provides extensive cross talk between the apoptotic pathway, and the other *NF-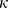
B*, and *JNK* pathways that also emanate from *TNF-R*. Extrinsic apoptosis occurs by means of the interaction between with the family of TNF receptors (we know from the gene expression analysis -see DEG-DB- that, for instance *TNFRSF21* is down regulated in Cas II-gly treated HeLa cells [Cas/HeLa]) with FADD (whose expression is down regulated in Cas/HeLa) and caspase-8 (low mRNA expression levels in Cas/HeLa). Protein is absent in CHP-212 and in Cas/HeLa. These facts altogether seem to pinpoint to the extrinsic route to apoptosis (also a significantly altered Reactome pathway, as is seen in Table 2) being somehow *turned off* in Cas/HeLa. However, Death receptor signaling, may also lead to mechanisms for intrinsic apoptosis. Molecules such as *Bid* are overexpressed in Cas/HeLa, and their negative regulators (like the family of *Bcl-2*-like molecules and *Bcl-2* associated athanogenes which enhance the anti-apoptotic effect of Bcl-2) are down-regulated in Cas/HeLa, while *cyt C* is over expressed and its antagonists like the cytochrome oxidases *PET117* and *SCO2* are sub-expressed in Cas/HeLa. Hence these patterns trace a route in the Death receptor signaling pathway that seem to imply the presence of intrinsic apoptosis and the formation of the apoptosome body.

**Table 2:** Statistically Significant Enrichment of Reactome Pathways for differentially expressed genes in HeLa cells treated with Cas II-Gly.

Reactome Pathways	FDR-corrected p-value
TNF signaling	0.00010306074036
Death Receptor Signalling	0.00032182163892
Extrinsic Pathway for Apoptosis	0.00032182163892
Cell Cycle	0.00092675401503
Iron uptake and transport	0.00348947088203
p53-Dependent G1 DNA Damage Response	0.00685024006157
p53-Dependent G1/S DNA damage checkpoint	0.00685024006157
G1/S DNA Damage Checkpoints	0.00759577614037
DNA Replication	0.00792865988103
M Phase	0.01655971283802
G1/S Transition	0.02195449920956
Cell Cycle Checkpoints	0.02798819809013
Cell Cycle, Mitotic	0.03213398331435
Mitotic G1-G1/S phases	0.03218601488173
Diabetes pathways	0.03266728941899
Apoptosis	0.03866643334412

Statistical significance was assessed by hypergeometric tests, corrected with the false discovery rate (FDR) algorithm.

#### IPA knowledge base analysis

**Figure 4:**
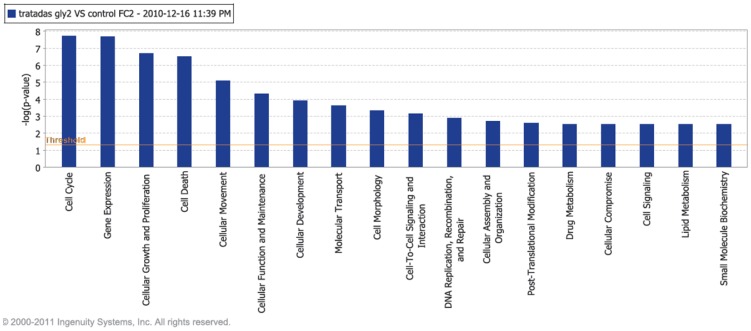
Main biological processes affected in HeLa cells treated with Cas II-gly. Image based in an analysis of the gene expression matrix in the Ingenuity® database for Systems Pathways Analysis (IPA) [28].

**Figure 5:**
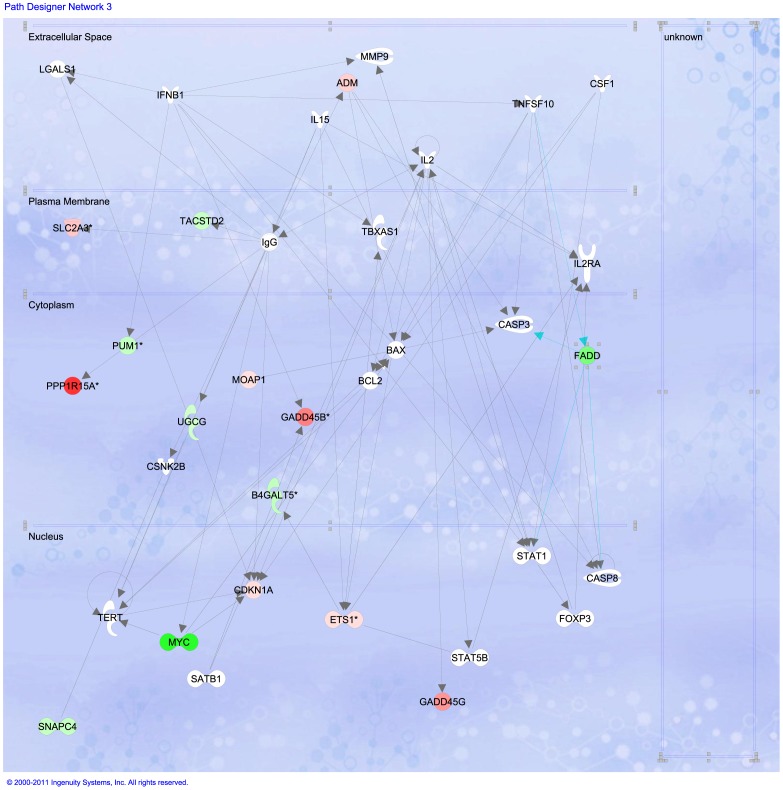
FADD signaling in the caspase pathway is affected in HeLa cells treated with Cas II-gly. Image based in an analysis of the gene expression matrix in the Ingenuity 
database for Systems Pathways Analysis (IPA) [28]. Molecules in green are transcriptionally down-regulated whereas molecules in red are transcriptionally over-expressed.

By using the Ingenuity Pathway Analysis tool we were able to study detailed pathways, correlated with their corresponding expression patterns. This allow us to see biochemical routes whose participant molecules present differential expression within our experiments. Figure 4 depicts the main processes with altered expression patterns in Cas/HeLa as compared with controls. Continuous lines inidicate that a gene is activated (repressed) only when the other gene is over-expressed (under-expressed), lines with an arrow point represent transcription factor interactions; broken lines indicate indirect gene interactions, blue lines/arrows represent that one gene interacts with other genes in the same pathway (e.g. FADD in figure 5).

As in the previous cases, these are related with alterations in cell cycle, growth and proliferation, cellular death, DNA repair, among others. For the relevance to test our hypothesis about the mechanism of action of Cas II-gly in HeLa cells, we choose to present a closer look of certain pathways. Figure 5 displays FADD signaling around some molecules involved in triggering apoptotic cascades. We can notice that the expression levels of certain genes is significantly altered (red = over-expressed, green = under-expressed, for gene-specific expression levels please refer to the DEG-DB table available at http://genomicacomputacional.inmegen.gob.mx/ehernandez/Casio/Casio_report.html) in Cas/HeLa as compared to controls (color intensity is proportional to the magnitud of the fold-change). We can notice how, at the cytoplasmic levels, down-alterations of membrane-related molecules *PUM1*, *UGCG* are present, as well as in molecules related with TNF-signaling such as *FADD* may imply membrane instabilities. In the other hand, there is a clear up-regulation of *PPP1R15A* and *GADD45B* that trigger the apoptotic program in response to DNA damage. At the nuclear level, we observe up regulation of *GADD45A* (a stress-response molecule) as well as a down regulation of *MYC* (which is a negative regulator of apoptosis), point out to stress-induced apoptosis initiation.

**Figure 6:**
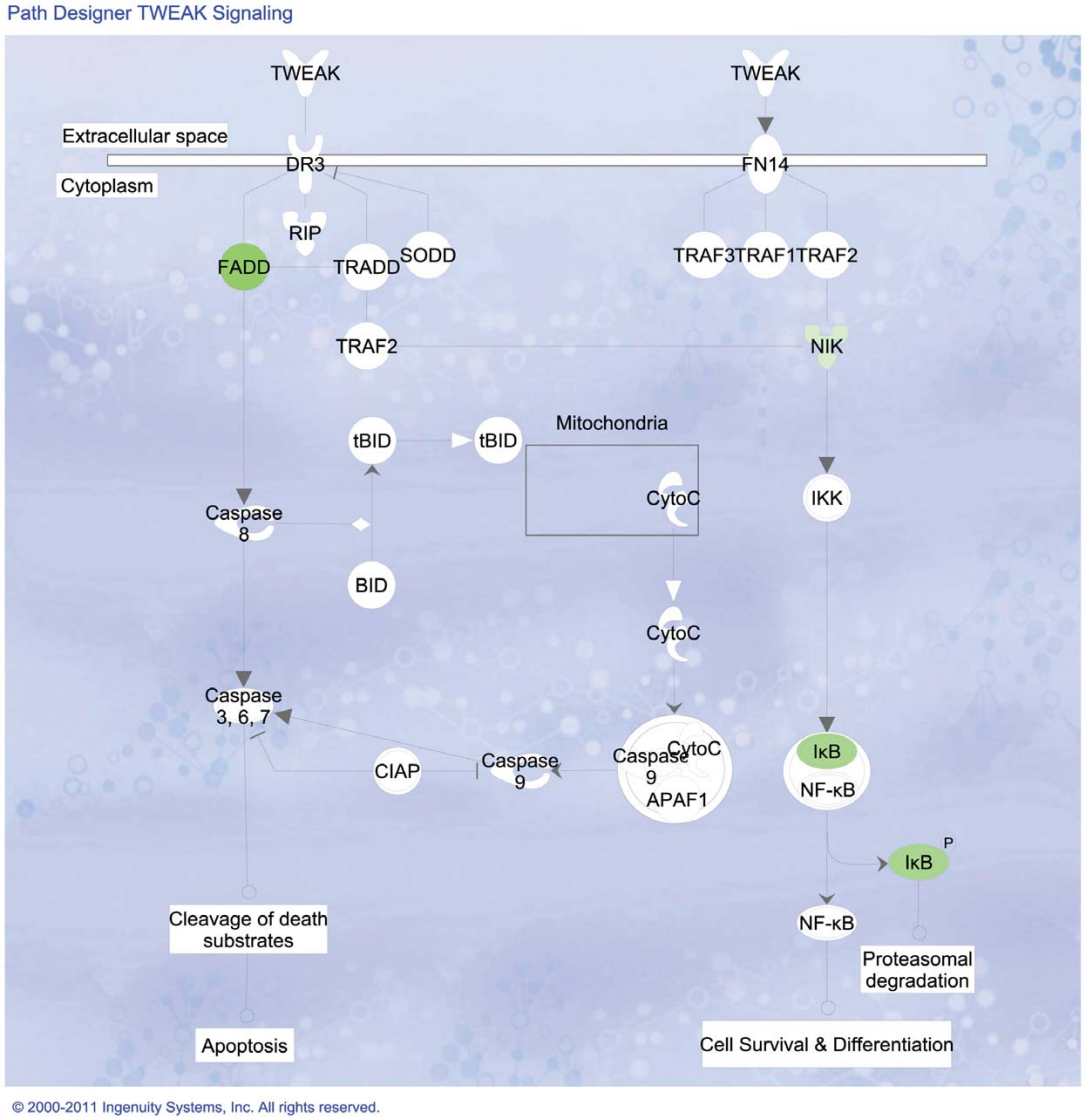
Response to reactive oxygen species pathway is affected in HeLa cells treated with Cas II-gly favoring apoptosis rather than differentiation and proliferation. Image based in an analysis of the gene expression matrix in the Ingenuity 
database for Systems Pathways Analysis (IPA) [28].

**Figure 7:**
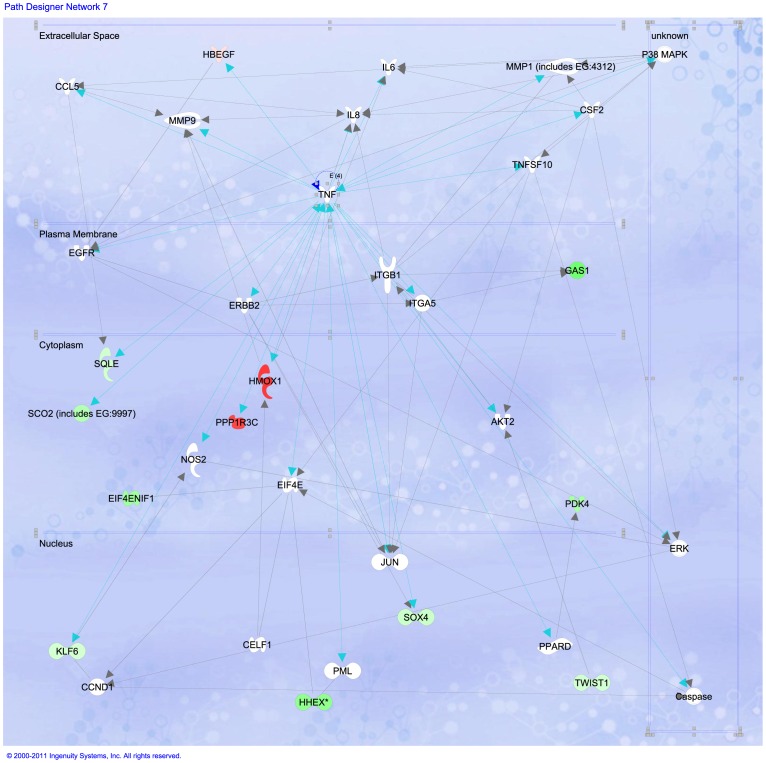
TNF signaling is affected in HeLa cells treated with Cas II-gly favoring apoptosis rather than differentiation and proliferation. Image based in an analysis of the gene expression matrix in the Ingenuity® database for Systems Pathways Analysis (IPA) [28]. Molecules in green are transcriptionally down-regulated whereas molecules in red are transcriptionally over-expressed. Notice the abundance of molecules -such as HMOX1- responding to oxidative stress.

A closer look at *FADD* signaling in the cytoplasmic region is depicted in Figure 6. *FADD* sub-expression blocks-out the initiation of extrinsic apoptosis *via caspase-8*; whereas *NIK* (also named *MAP3K14* which is an NF-kappa-beta-inducing kinase) and *IKB* (or *NFKBIB*) sub-regulation may inactivate, both proteasomal degradation and cell survival processes thus indirectly leading to cell death, likely *via* intrinsic apoptosis. The initiation phase seem to be started by response to oxidative stress. In order to take a closer look of these processes, we will examine TNF signaling responses to stress in Figure 7. We can recall large over-expression of *HMOX1* and *PPP1R3C* (which activates *p53*, driving *p21* and initiating cell cycle arrest and apoptosis). Down-regulation of *GAS1* (growth arrest specific factor 1), *SCO2*, *KLF6*, *TWIST1*, *SOX4* and the homeobox *HHEX*, all of them participating in differentiation and cell fate processes may indicate some evidence of cell cycle arrest.

#### Gene set enrichment analysis

Statistical enrichment analysis of our gene expression patterns *versus* a database of molecular signatures for cancer modules [40]. Results for the top 20 modules (out of 105 statistically significant hits) are shown in Table 3. We can notice that gene expression patterns in Cas/HeLa correspond with molecular signatures that have been related with processes such as metabolic detoxification, functionalization of compounds, biological oxidation, *cyt C* activity and other functions related to cytotoxicity. Also processes related to DNA damage such as p53, NOD and VEGF signaling, as well as p38 MAPK activation are enriched. Acute inflammatory response, chemokine signaling, PI-3K cascading and stress induction of HSP’s are trademarks of responses to stress, and in particular of oxidative stress may as well seem to point out to Cas II-gly-induced stress and cytotoxicity. For details of GSEA cancer gene set modules (genes and their expression fold changes in each module) please refer to http://genomicacomputacional.inmegen.gob.mx/ehernandez/Casio/Casio_GSEA_modules.xls.

**Table 3:** Top 20 GSEA - Cancer Modules for differentially expressed genes in HeLa cells treated with Cas II-Gly.

Module	FDR corrected p-val	Relevant features
MODULE_235	0.006254237	Phase II conjugation - metabolic detoxification
MODULE_164	0.003127119	p53 signaling pathway,
MODULE_6	0.007922776	Cell adhesion, functionalization of compounds
MODULE_247	0.020577544	Genes involved in Biological oxidations
MODULE_488	0.020014847	Cell Cycle: G1/S Check Point, local acute inflammatory response
MODULE_263	0.018957132	NOD-like receptor signaling pathway (NF-KB related)
MODULE_108	0.022294335	Selective expression of chemokine receptors during T-cell polarization
MODULE_178	0.028853938	Genes involved in PI-3K cascade
MODULE_362	0.026101848	Genes involved in Signaling by VEGF
MODULE_444	0.028056284	Genes involved in SHC-mediated cascade, regulation of cell adhesion
MODULE_14	0.026060386	Cytokine Network
MODULE_29	0.02646123	GTP hydrolysis
MODULE_516	0.025891503	Regulation of cell adhesion
MODULE_83	0.026049469	Cytochrome c oxidase activity
MODULE_76	0.02518265	Toll-like receptor signaling pathway, NOD-like receptor signaling pathway
MODULE_462	0.02532188	Genes involved in Biological oxidations, Phase II conjugation - metabolic detoxification
MODULE_151	0.02696601	Oxidoreductase activity, mitochondrial membrane, cytochrome c oxidase activity
MODULE_355	0.025467897	Stress Induction of HSP Regulation, p38 MAPK Signaling Pathway
MODULE_93	0.02453315	Oxidoreductase activity, metal ion transporter activity, glutathione peroxidase activity
MODULE_5	0.032609396	Response to stress, inflammatory response, protein kinase activity

Statistical significance was assessed by hypergeometric tests, corrected with the false discovery rate (FDR) algorithm.

### Functional Analysis in HeLa and Comparison with CHP-212

**Figure 8:**
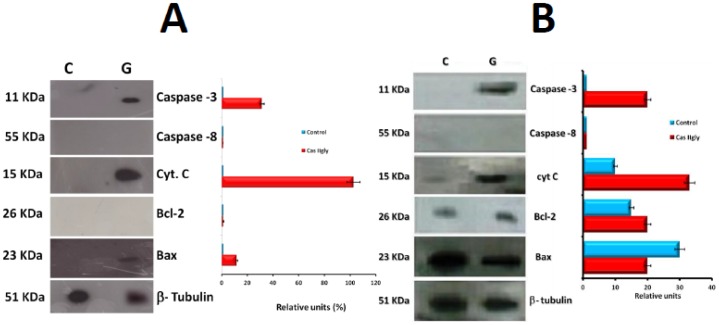
Western Blot for molecules of interest in HeLa and CHP-212. Cells were previously treated with Cas II-gly at their corresponding IC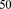, C = control cells without treatment, G = Cas II-gly treated cells. Western blot was done in the cytosolic portion after 6 h treatment for HeLa (panel A) and 2 h of treatment for CHP-212 (panel B), in order to detect active *caspase-3*, *caspase-8* cleaved, *cyt C*, *Bcl-2* and *Bax*. Protein expression was normalized to the 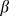-tubulin loading control. Each bar represents the mean 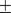 SD (P
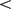
0.05) from three independent experiments and expressed as Relative units measured by percentage of optical densitometry (% OD).

**Figure 9:**
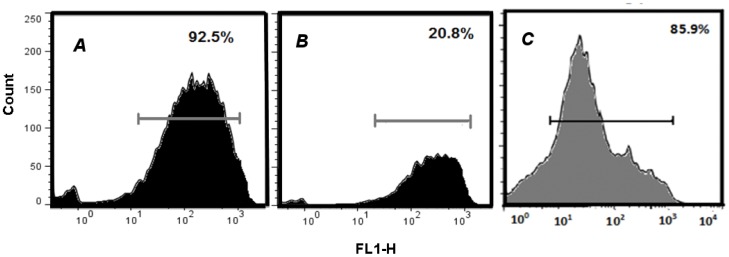
Mitochondrial activity as determined by MitoTracker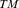 in HeLa and CHP-212. Mitochondrial activity was measured by accumulated MitoTracker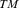 green fluorescence in mitochondria by means of flow cytometer (FL1-H channel). In all cases the bar represents the fluorescence emission of the dye; y-axis corresponds to the quantity of cells and data is representative of three independent experiments. Panel A corresponds to untreated control cells, Panel B to Cas/HeLa and Panel C to Cas/CHP-212.

The half-maximal inhibitory concentrations (IC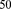) were different in HeLa and CHP-212. After subcellular fractionation, the cytosolic apoptotic components were identified in all cases (Figure 8). We found that *caspase-8* was absent in both, cervix-uterine (HeLa) cells and in NB (CHP-212), and *caspase-3* was active in all treated cells (40% in HeLa and 20% in CHP-212). Other evaluated molecule was *cyt C* released from mitochondria (102% in HeLa and 33% in CHP-212). The antiapoptotic molecule *Bcl-2* was absent in HeLa, while it was present in both CHP-212 cells (Control 15%, treated with Cas II-gly 20%). Finally the evaluation of the proapoptotic molecule *Bax*, was present in HeLa and CHP-212 treated cells (around 20% in both cases). Interestingly enough *Bax* was absent in HeLa control cells while it was present in CHP-212 controls (30%). The disturbance of 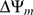 reflects a degree of mitochondrial damage by Cas. Mitochondrial activity was monitored by means of MitoTracker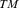. Significant degree of mitochondrial activity was detected in the control cells (92.5%), is strongly diminished in HeLa Cells (20.8%) but just slightly diminished in CHP-212 (85.9%) (Figure 9).

**Figure 10:**
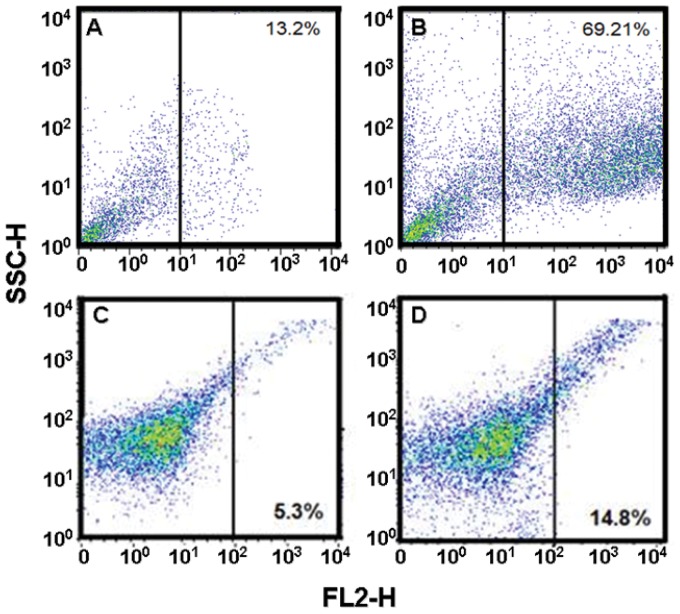
AmplexRed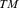 peroxide determination by flow cytometry in HeLa and CHP-212. The quantity of cells expressing Hydrogen peroxide was detected with AmplexRed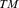 by flow cytometry in the FL2-H channel, whereas SSCH corresponds to side scatter channel. Panel A: untreated HeLa cells, Panel B: Cas/HeLa, Panel C: untreated CHP-212 cells, panel D: Cas/CHP-212. Positive controls corresponds to 3 h UV-irradiated cells. Data is representative of three independent experiments for each experimental condition.

**Figure 11:**
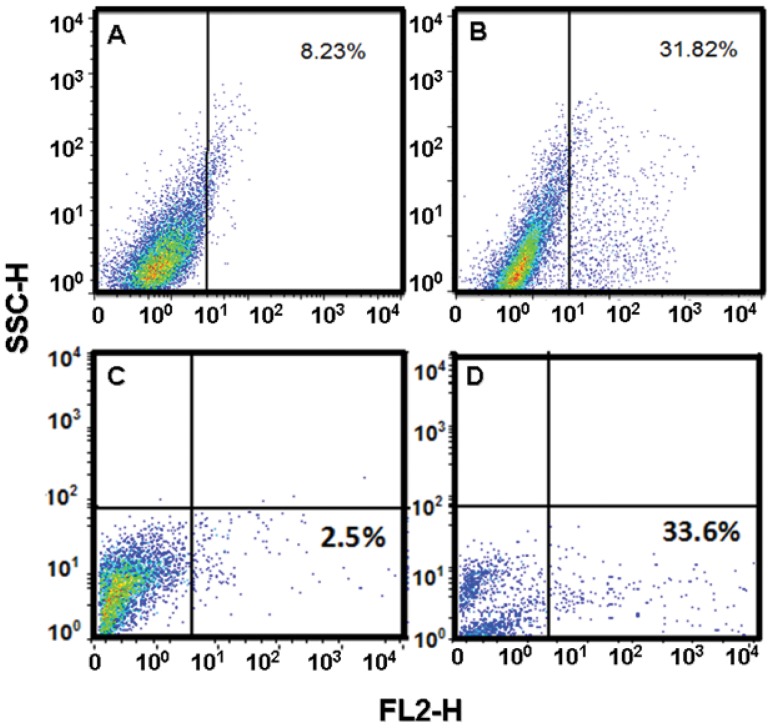
MitoSox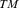 superoxide determination by flow cytometry in HeLa and CHP-212. Superoxide radical was measured by flow cytometry with MitoSox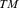 (FL2-H channel) dye; SSCH corresponds to side scatter channel. Panel A: untreated HeLa cells, Panel B: Cas/HeLa, Panel C: untreated CHP-212 cells, panel D: Cas/CHP-212. Positive controls, are 3 h UV-irradiated cells. Data is representative of three independent experiments for each experimental condition.

**Figure 12:**
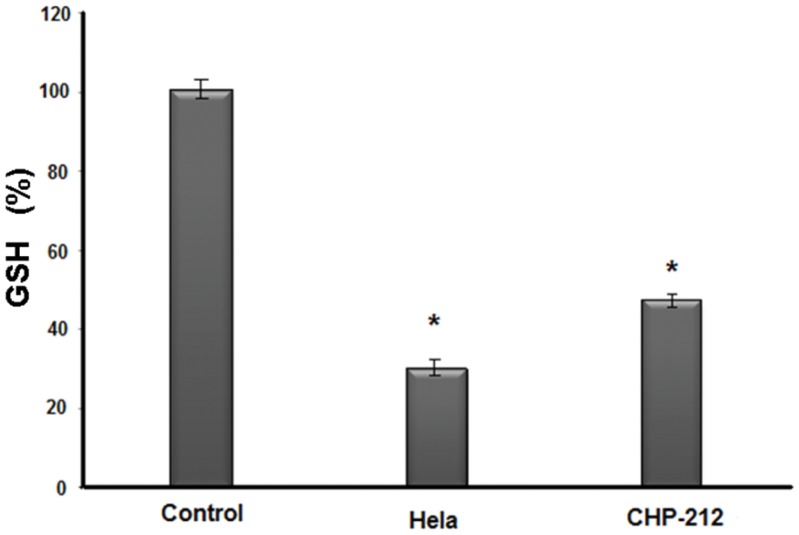
Glutathione determination by ELISA in HeLa and CHP-212. GSH intracellular index was measured by accumulated MCB fluorescence in a microtiter plate reader at 380/460 nm. GSH in non-treated cells was considered as a normalizing top value (100%); whereas negative control cells were MCB plus Cell Lysis Buffer. Data represent means 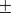 standard deviation of three wells and experiments were replicated once. Differences between groups were assessed (marked with an asterisk) by ANOVA analysis with significance at P
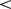
0.05.

We found an important quantity of cells with ROS expression. All reactive species were detected by flow cytometry. Peroxide expression (Figure 10 as determined by AmplexRed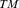, see methods) resulted greatly increased in HeLa cells (13.2% in control, 69.21% in Cas/HeLa) and less dramatically in CHP-212 (5.3% in control, 14.8% in Cas/CHP-212). It is likely that this increase is, at least partially, a consequence of a higher time of exposure in HeLa cells. Superoxide expression was observed as a cellular response to treatment with MitoSox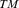 (Figure 11). In such experiment we observed similar expression levels for both cell lines, being (8.23% in control, 31.82% in Cas/HeLa) and (2.5% in control, 33.6% in Cas/CHP-212). In order to assess ROS activation by Cas II-gly treatment, we also determined the intracellular GSH average concentration by MCB dye. GSH in non-treated cells was considered as a normalizing top value (100%). In HeLa treated cells, intracellular GSH level decreased to a value of around 35%, whereas GSH level in CHP-212 cells diminished only to about 52% (Figure 12).

### Quantitative PCR Validation of Selected Genes

**Figure 13:**
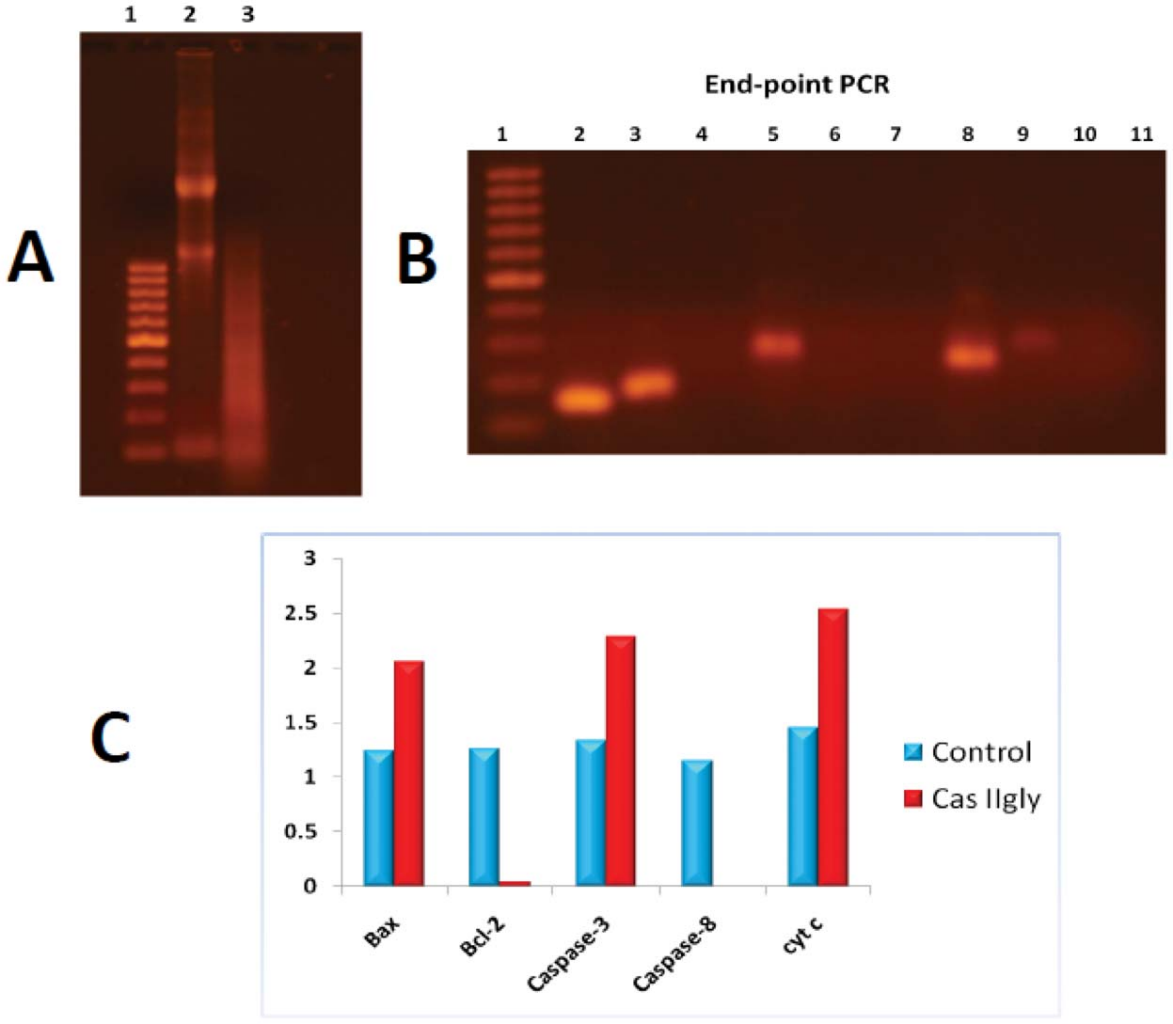
qPCR validation of gene expression for selected genes relevant to intrinsic apoptosis. Panel A presents results for total RNA integrity: Band 1 = Molecular weight marker, Band 2 = HeLa control (no-treatment), Band 3 = HeLa treated with Cas IIgly (40 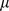-M, 6 h). Panel B display endpoint qPCR: Bands 1: Molecular weight marker, 2: Bax (HeLa control); 3: Bcl-2 (HeLa control); 4: caspase-8 (HeLa control); 5: cyt C (HeLa control); 6: caspase-3 (HeLa control); 7: caspase-3 (HeLa Cas IIgly); 8: cyt C (HeLa Cas IIgly); 9: Bax (HeLa Cas IIgly); 10: Bcl-2(HeLa Cas IIgly); 11: caspase-8 (HeLa Cas IIgly). Panel C quantitative PCR levels for cases (red) and controls (blue)(for numerical values please refer to Table 4). qPCR assessment used HPRT1 as a control gene.

As described in methods, qPCR validation experiments were performed in selected genes (Bax, Bcl-2, Caspase-3, Caspase-8, and Cytochrome C). Such genes were selected to validate the hypothesis of intrinsic apoptosis driven by ROS and oxidative stress as the main mechanism of action of Casiopeína II-gly in HeLa cells. As shown in Figure 13 results were consistent with those of whole genome microarray expression data and were also consistent with Western blot experiments. In Figure 13 panel A we can see results for RNA integrity, in panel B we present end-point PCR results, also displayed as a bar-plot in panel C. Numerical values of such results are presented in Table 4. One can see that microarray data and qPCR point out to over-expression of Bax, Caspase-3 and Cyt C, as well as Bcl-2 and Caspase-8 down-regulation (even inhibition) in cases (likely by the action of the treatment). The presence of high levels of Caspase-3 and Bax (also seen as the associated proteins, see Figure 8 panel A) point out to mitochondrial (or intrinsic apoptosis), whereas the abscense of Caspase-8 rule-out (to a certain extent) the possibility of extrinsic apoptosis. High levels of Cyt C may indicate the presence of oxidative stress, a phenomenon that is also consistent with the results of whole genome expression analysis and its associated statistical and data-mining analyses, as well as with western blot experiments.

**Table 4:** qPCR expression levels for genes involved in apoptosis.

Gene	Control	Cas-II gly
Bax	1.24	2.05
Bcl-2	1.26	0.045
Caspase-3	1.33	2.28
Caspase-8	1.15	0.002
Cyt C	1.45	2.53

Values are indicative of over-expression of Bax, Caspase-3 and Cyt C and sub-expression of Bcl-2 and Caspase-8 in cells treated with Cas-II gly in agreement with the results of whole genome microarray gene expression analysis.

## Discussion

Metabolic drugs have recently emerged as a suitable alternative therapeutic approach against malignant chemo- and radiotherapy-resistant tumors. The Copper-based drug Cas II-gly has shown its ability to promote DNA fragmentation and apoptosis in a process mediated by reactive oxygen species in a number of tumor cells tumor cells [6], [9], [15], [18], [41]. In this sense, it has been described that some metals such as Copper and some complexes of them, are cytotoxic due to their high potential to participate in redox reactions. This event generates ROS, including hydrogen peroxide (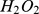), and radicals hydroxyl (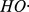) and superoxide (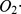) which could be responsible of the programmed cell death. Currently, some efforts to fight these cancers are focused on improving our understanding of the apoptotic phenomenon.

Apoptosis is characterized by morphological and biochemical events such as the activation of caspases, chromatin condensation and rearrangement at the nuclear periphery, as well as DNA fragmentation, cell shrinkage, and the formation of apoptotic bodies [15]. Cell death induced by Cas II-gly is concomitant with ROS increase [15], [41] and the mechanisms involved could be related to Cas II-gly capability to activate 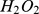 by a Fenton-like reaction using naturally occurring cell reducing agents as the source of electrons [19], [41]. On the other hand, it is well known that Copper can react with GSH to form stable complexes [42], [43], which are still redox active [44]. In both cases, these mechanisms of action might lead to a decline of GSH levels. Additionally, DNA damage and mitochondrial dysfunction also contributes to the increase of ROS levels and therefore, to GSH depletion. The mechanism by which GSH interacts with metals is extremely significant in metal toxicity and it is directly reflected in intracellular GSH concentrations.

As we just said, the precise mechanism of action for Cas II-gly is still poorly understood and a detailed description of the events that lead to cell death remains unexplained. This study further explores the mechanism of Cas II-gly [15] inducing apoptosis by ROS in neuroblastoma and cervix-uterine cell lines. Metal-based chemotherapeutic research is increasing [45]. In particular, studies began to be conducted on the efficacy of Casiopeínas. Casiopeína II-gly belongs to a coordination family compounds with a Copper core that has been shown an antiproliferative and antitumoral effects in tumors such as neuroblastoma [18], medulloblastoma [9], glioma [6], breast, cervix-uterine, lung, and colon [19]. Although their action mechanism is still poorly understood, it has been suggested that they are able to induce ROS expression, to bind DNA by adenine and thymine interactions or to block oxidative phosphorylation.

Regarding Cas II-gly action mechanism, it has been demonstrated that interferes both the electron transfer chain and oxidative phosphorylation in mitochondria isolated from cardiac muscle [12] and glioma cells [6], and also affects mitochondrial ATP synthesis in rat AS-30D hepatocarcinoma and HeLa cells, two highly OxPhos-dependent tumors [46], by interacting with the reactive thiol groups of Krebs cycle (pyruvate, 2-OG, succinate) dehydrogenases [11]–[13]. Cas II-gly is able to produce oxidative damage which in turn is a consequence of the lower intracellular glutathione content [15], [18], [41] but it is successfully abrogated by the cell’s naturally occurring antioxidant defences, such as GSH.

Oxidative stress is a putative mediator of apoptosis through many different mechanisms as 1) the intracellular increase of ROS [47] or the depletion of endogenous antioxidants [48]; 2) the action of some antioxidants-such as N-acetyl-L-cystein (NAC)-which act as intracellular ROS scavengers, thus inhibiting the activation of caspases [49]; 3) the overexpression of Mn superoxide dismutase (SOD), which restores the mitochondrial transmembrane potential, thus inhibiting apoptosis [49], [50]; 4) the overexpression of Cu/Zn-SOD, which delays apoptosis by scavenging O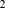 [51]; and 5) the overexpression of the mitochondrial phospholipid hydroperoxide, glutathione peroxidase (GPx) that inhibits the generation of ROS formation.

Cas II-gly may induce apoptosis without DNA oligonucleosomal fragmentation in B cells lymphoma line (CH1). This event reveals a relatively low level of caspase activation that was confirmed with the inhibition of apoptosis by ZVAD-FMK, a wide broad caspase inhibitor [16]. At low concentrations (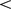
10 nM), Cas II-gly inhibits the rates of state 3 and uncoupled respiration of mitochondria isolated from the rat liver, kidney, heart, and AS-30D hepatoma [12]. When concentrations higher than 10 nM, this compound stimulates basal respiration, followed by its inhibition, a K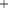-dependent swelling, collapse of membrane potential, and late *cyt C* release in rat liver, kidney, heart, and AS-30D hepatoma [12]. In glioma C6 cells treated with low Cas II-gly concentrations (1 or 2.5 g/ml) apoptosis was ROS-independent. Whereas at higher concentrations (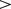
5 g/ml) of Cas II-gly, was observed *AIF* translocation and *caspase-3* activation that imply apoptosis ROS-dependent or -independent what was assured by 20 nM NAC [6].

We found that treatment with Cas II-gly in cervix-uterine and neuroblastoma tumors may be able to develop mitochondrial apoptosis. We detected the expression of different apoptotic molecules, in particular those related with the intrinsic route to apoptosis. We measured *caspase-3* expression in both cell lines, confirming the apoptotic phenomenon. Both HeLa and CHP-212 cells may present mitochondrial or independent of caspases apoptosis, which we conclude is due to the absence of *caspase-8* expression. Another apoptotic molecule which is abundant in the cytoplasmic fraction of both cell lines (remarkably higher in HeLa cells) is *cyt C* also part of the mitochondrial route. Anti-apoptotic *Bcl-2* expression is very low in NB and absent in cervix-uterine tumors. Apoptotic molecule *Bax* is expressed in both cell lines. Detoxification of the ROS system was measured by means of GSH enzyme. Thus, we observed that it was more active in HeLa than in NB cells.

**Figure 14:**
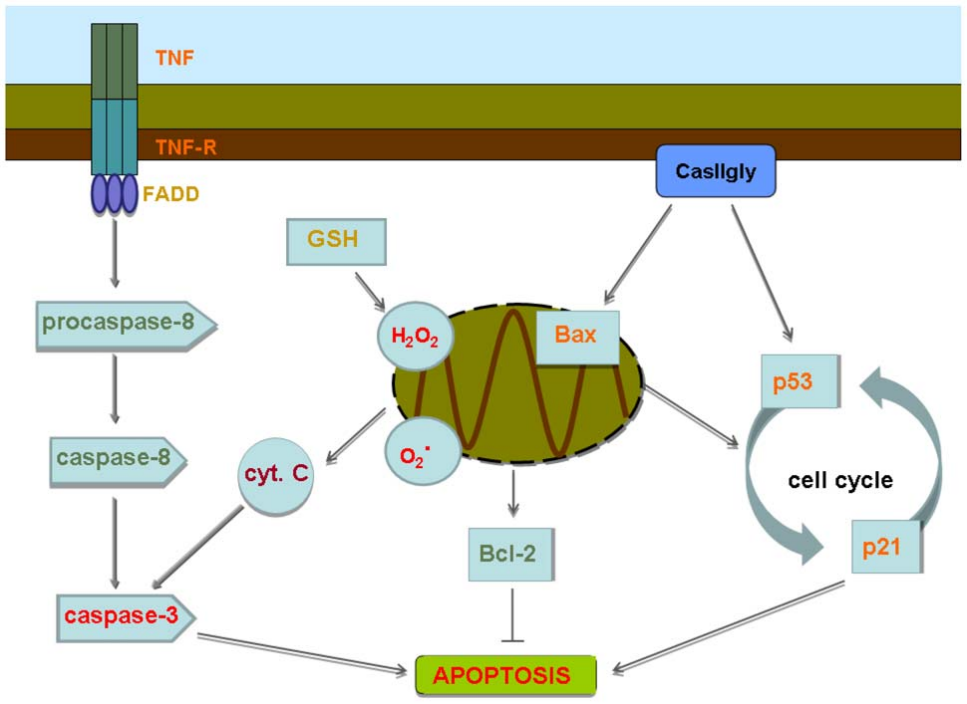
A model for Cas II-gly-affected apoptotic pathways. Depicted are the pathways that may be affected by the action of Cas II-gly. Molecules are tag-name color-coded: red color corresponding to over-expression and green color to sub-expression, other colors describe intermediate states. We can notice that the intrinsic route to apoptosis is favored by *caspase-3* activation via *cyt C* over-expression, high ROS concentrations, as well as cell cycle arrest; whereas the extrinsic route to apoptosis is somehow diminished due to very low-expression of *pro-caspase-8* and *caspase-8*. Anti-apoptotic processes are switched-down due to low levels of *Bcl-2*.

If we also recall the results of whole-genome gene expression analysis, higher over-expression of *BCL-10*, may activate *caspase-9* hence enabling interactions with *TNF* concurrent with over-expression of *NDRG1*, a known *p53*-mediated caspase activator *via* the intrinsic route, while molecules associated with the extrinsic route such as *FADD* and *FZDZ* appear under-expressed. If we also notice over expression of *MOAP1* and *DEDD2*, this may imply an overall reduction of *TNFR1*-signaling, resulting in diminished apoptotic extrinsic pathway activity. Over-expression of *HMOX1* and *GADD45G* results in moderate up-regulation of *MT1X* (that regulates metals and molecules with free radicals) concomitant with oxidative stress. In view of all these, we can conclude that the pathway favored by Cas II-gly in HeLa and CHP-212 cells is the intrinsic route. A finding supported by the absence of *caspase-8*, the presence of *caspase-3*, with *cyt C* coming from mitochondria, followed by entry of *Bax* to mitochondria, which in turn is releasing 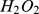 and 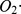 thus creating a favorable micro-environment for apoptosis. If we refer to Figure 14, we can see molecules with their tag-names color-coded: red color corresponding to over-expression and green color to sub-expression, other colors describe intermediate states. The branch corresponding to the extrinsic route to apoptosis starts with medium to moderately high expression of *TNF* and its receptor with a somehow lower expression of *FADD*. However, small expression of both, *pro-caspase-8* and *caspase-8* make quite difficult for the cells to undergo extrinsic apoptosis. In the other hand, the presence of high concentrations of reactive oxygen species 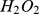, 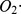, as well as *cyt C* lead to *caspase-3* activation (*caspase-3* is also present in high quantity) thus enabling the intrinsic route to apoptosis. Apoptosis is also enabled by cell cycle arrest driven by moderately high expression of *Bax*/*p53*/*p21*. As we have shown, this hypothesis is supported by whole-genome gene expression experiments, by statistical analysis and data mining; and by functional tests (Western blots, Elisa, Flow cytometry, etc.). Additional arguments are given in Tables 1, 2, and 3 as a result of computational analysis based in gene expression patterns.

Of course, there are still a number of features to be unveiled and hypothesis to be tested in relation with the chemotherapeutics of apoptosis induction by Copper compounds (Casiopeínas). Apoptosis is known to be a quite complex phenomenon with a lot of pathway cross-linking [52], [53]. The set of experimental and computational-statistical analyses here presented point-out to mitochondrial apoptosis as the favored route to programmed cell death due to the action of Casiopeína II-gly in HeLa cells as well as in CHP-212. Much work has still to be done, however in order to establish the validity and feasibility of these model in these and other cell lineages.
